# Regulation of ubiquitin-proteasome and autophagy pathways after acute LPS and epoxomicin administration in mice

**DOI:** 10.1186/1471-2474-15-166

**Published:** 2014-05-22

**Authors:** Cécile Jamart, Aldrin V Gomes, Shannamar Dewey, Louise Deldicque, Jean-Marc Raymackers, Marc Francaux

**Affiliations:** 1 Institute of Neuroscience, Université catholique de Louvain, Place Pierre de Coubertin, 1 bte L8.10.01, Louvain-la-Neuve 1348, Belgium; 2 Department of Neurobiology, Physiology & Behavior, University of California, Davis, CA 95616, USA; 3 Exercise Physiology Research Group, Department of Kinesiology, KU Leuven, Leuven B-3001, Belgium

**Keywords:** MuRF1, MAFbx, LC3, Endotoxemia, Proteasome inhibitors

## Abstract

**Background:**

The ubiquitin-proteasome pathway (UPP) is a major protein degradation pathway that is activated during sepsis and has been proposed as a therapeutic target for preventing skeletal muscle loss due to cachexia. Although several studies have investigated the modulation of proteasome activity in response to LPS administration, none have characterized the overall UPP response to LPS administration in the fate of proteasome inhibition.

**Methods:**

Here, we determined the modulation pattern of the main key components of the UPP in the gastrocnemius (GAS) of mice during the acute phase of lipopolysaccharide (LPS)-mediated endotoxemia (7.5 mg/kg – 8 h) by measuring all three β1, β2 and β5 activites of the 20S and 26S proteasomes, the levels of steady state polyubiquitinated proteins, mRNA levels of muscle ligases, as well as signaling pathways regulating the UPP. Another goal was to assess the effects of administration of a specific proteasome inhibitor (epoxomicin, 0.5 mg/kg) on UPP response to sepsis.

**Results:**

The acute phase of LPS-induced endotoxemia lowered GAS/body weight ratio and increased *MuRF1* and *MAFbx* mRNA concomitantly to an activation of the pathways known to regulate their expression. Unexpectedly, we observed a decrease in all 20S and 26S proteasome activities measured in GAS, which might be related to oxidative stress, as oxidized proteins (carbonyl levels) increase with LPS. While significantly inhibiting 20S and 26S proteasome β5 activities in heart and liver, epoxomicin did not lower proteasome activity in GAS. However, the increase in mRNA expression of the muscle ligases *MuRF1* and *MAFbx* were partially rescued without affecting the other investigated signaling pathways. LPS also strongly activated autophagy, which could explain the observed GAS atrophy with LPS-induced reduction of proteasome activity.

**Conclusions:**

Our results highlight an opposite regulation of UPP in the early hours of LPS-induced muscle atrophy by showing reduced proteasome activities and increased mRNA expression of muscle specific ligases. Furthermore, our data do not support any preventive effect of epoxomicin in muscle atrophy due to acute cachexia since proteasome activities are not further repressed.

## Background

Skeletal muscle loss due to cachexia is clinically distinct from other forms of muscle atrophy such as muscle disuse or sarcopenia because it is always associated with an underlying disease and inflammation [1]. Endotoxic shock - or sepsis - induces a severe and acute form of cachexia, mainly due to an increase in protein degradation [2]. Endotoxic shock also induces hypotension, vascular damages and inadequate tissue perfusion that lead to multiple organ failure, including heart and liver failure [3]. The pathogenesis of sepsis depends predominantly on lipopolysaccharide (LPS), which is a membrane component of gram negative bacteria responsible for the endotoxic activity [4]. Therefore, administration of LPS to animals has been extensively used to mimic sepsis [5].

The ubiquitin proteasome pathway (UPP) is a major protein degradation pathway that is modulated during sepsis. Briefly, ubiquitin-proteasome-dependent proteolysis is a two-step ATP-consuming process. In a first step, the substrate is covalently bound to a polyubiquitin chain through the sequential action of a triplet of enzymes. The second step implies the recognition, unfolding and degradation of the polyubiquitinated substrate by a multicatalytic complex, namely proteasome 26S. Polyubiquitin chain formation requires the sequential action of three types of ubiquitin enzymes, respectively known as ubiquitin-activating (E1), ubiquitin-conjugating (E2) and ubiquitin-ligase (E3) enzymes. In catabolic conditions, the most important regulations occur at the level of E3s, which are numerous and responsible for the specificity of protein-substrate recognition. In skeletal muscle cells, upregulation of the tissue-specific E3 ligases muscle ring finger 1 (*MuRF1*) and muscle atrophy F-box (*MAFbx*) in response to LPS administration is now well established [6,7].

Several forms of proteasomes exist in cells. The main form, called the 26S proteasome, is composed of the 20S core associated with one or two 19S particles that contain subunits with ATPase activities. Another form is the 20S proteasome, which is ATP-independent but capable of degrading oxidized proteins [8,9] as well as certain non-ubiquitinated proteins [10,11], while the 26S form is responsible for degrading polyubiquitinated proteins. Proteolytically active sites are localized in the β1, β2 and β5 subunits of the 20S proteasome. They are described as caspase-like, trypsin-like and chymotrypsin-like activities, respectively, in accordance to the peptide bonds that they cleave preferentially [12]. The β5 activity is believed to be the rate-limiting step for polypeptide degradation by the proteasome [12].

Several authors have suggested that proteasome could be a potential therapeutic target for preventing the consequences of sepsis [3,13,14]. Among the drugs designed for that purpose (for review see [15]), epoxomicin is a natural product that was initially isolated from an *Actinomycetes* strain for its antitumor properties. Unlike most proteasome inhibitors, epoxomicin acts specifically and does not inhibit nonproteasomal proteases. It irreversibly and covalently binds to the six 20S proteasome catalytic subunits, with higher affinity for the β5 subunit active site [16].

A few studies investigated the modulation of β5 proteasome activity in response to LPS administration [17-20]. However, we are unaware of any investigation characterizing the overall modulation pattern of the UPP in skeletal muscle by measuring all 20S and 26S β1, β2 and β5 activities, mRNA of muscle ligases, the levels of steady state polyubiquitinated proteins as well as signaling pathways regulating UPP during LPS-mediated endotoxemia. Recently, macroautophagy, here called autophagy, has been implicated in LPS-mediated muscle atrophy [21-23]. Unc 51-like kinase 1 (ULK1) plays an essential role in the initiation of autophagosome membrane formation. The subsequent elongation of this membrane is under the control of several autophagy related-gene (ATG) proteins, including microtubule-associated protein 1 light chain 3 (LC3). Finally, the mature autophagosome, whose membrane includes the lipidated form of LC3 (LC3II), fuses with lysosomes containing hydrolases such as cathepsins.

This study was undertaken to determine how the UPP is regulated in skeletal muscle during the acute phase of LPS-mediated endotoxemia. Given that several authors suggest that the proteasome itself could be a potential therapeutic target for preventing sepsis consequences [3,13,14], a second goal was to assess the effects of epoxomicin administration on the UPP response to sepsis.

Our studies show that, during the acute phase of LPS-induced endotoxemia, both *MuRF1* and *MAFbx* ligases are activated, together with the autophagy pathway. On the opposite, 26S and 20S proteasome activities are drastically reduced. This suggests that mechanisms of retro-feedback could occur inside muscle cell to prevent any excessive protein breakdown.

## Methods

### Ethics statement

This study was carried out in strict accordance with the Belgian Law of April 6, 2010 on the protection of laboratory animals. The protocol was approved by the Committee on the Ethics of Animal Experiments of the Université catholique de Louvain (agreement number LA 1220548). All efforts were made to minimize suffering.

### Experiment protocol

Eighteen male C57BL6 mice (15 weeks old) were obtained from the animal facility of the Université catholique de Louvain. Animals were housed at 22°C on a 12 h dark-light cycle with ad libitum access to food and water. The day before the experiment, mice were placed in individual cages. Mice were randomly assigned either to control (C, n = 6), LPS treatment (L, n = 6), or LPS plus epoxomicin treatment (L + E, n = 6) groups. On the day of experiment, mice were weighed and injected intraperitoneally with vehicle (10% DMSO in saline solution, C and L groups) or epoxomicin (Peptide Institute, Osaka, Japan), 0.5 mg/kg (L + E group). This dose was selected because it was reported to inhibit the proteasome but was nontoxic *in vivo*[16]. One hour after the first injection, animals were injected with vehicle (saline solution, C group) or LPS O127:B8 (Sigma Aldrich, Bornem, Belgium), 7.5 mg/kg (L and L + E groups). Mice had free access to water. As sepsis has been associated with anorexia [24], food was removed in each group immediately after the first injection to avoid any differences in food intake. Mice were anesthetized eight hours after LPS administration with a lethal injection of a mix of ketamine (200 mg/kg) and xylazine (20 mg/kg). This time point post-LPS was selected because it was previously shown to drastically increase *MuRF1* and *MAFbx* expression [7], which are considered as accurate markers of the atrophy process [25]. Before dissection, the depth of anesthesia was assessed by the absence of eyelid and pedal withdrawal reflexes. Animals were weighed, and the right and left gastrocnemius muscles as well as the heart and the liver were excised and quickly frozen in liquid nitrogen. Muscles were subsequently weighed and samples were stored at -80°C until further analysis.

### Protein extraction for immunoblotting

Muscles were crushed with mortar and pestle in liquid nitrogen. For each sample, one half of the powder was kept at -80°C for RNA extraction. The other part was homogenized in ice cold buffer containing 20 mM Tris, pH 7.0, 270 mM sucrose, 5 mM EGTA, 1 mM EDTA, 1% Triton X-100, 1 mM sodium orthovanadate, 50 mM sodium β-glycerophosphate, 5 mM sodium pyrophosphate, 50 mM sodium fluoride, 1 mM DTT (1,4-dithiothreitol) and a protease inhibitor cocktail containing 1 mM EDTA (Roche Applied Science, Vilvoorde, Belgium). Homogenates were centrifuged for 10 min at 10,000 g, 4°C. Supernatants were stored at -80°C. Protein content was determined using the DC protein assay kit (Bio-Rad, Nazareth Eke, Belgium) with bovine serum albumin (BSA) as a standard.

### SDS-PAGE and immunoblotting

Proteins (35 μg) were combined with Laemmli sample buffer and warmed for 5 min at 95°C before loading on gels. For protein carbonyl measurements, 5 μg protein were derivatised with 2,4-dinitrophenyl hydrazine before electrophoresis, as described by the protein oxidation kit from Merck Millipore (Billerica, MA, USA). Samples were separated by SDS-PAGE for 1 h at a constant intensity of 40 mA and transferred to PVDF membranes at 80 V for 2 h. Membranes were blocked 1 h in 0.1% Tween 20 Tris-buffered saline (TBST) and 5% non-fat dry milk, then incubated overnight at 4°C with one of the following primary antibodies: Regulatory particle triple-A ATPase 1 (RPT1; Enzo, NY, USA), dinitrophenyl (Sigma Aldrich), phospho-Akt ^Serine(Ser) 473^, total Akt, phospho-factor 4E binding protein 1 (4E-BP1) ^Threonine(Thr) 37/46^, eukaryotic elongation factor 2 (eEF2), phospho-forkhead box O3a ^Thr32^ (FoxO3a^Thr32^), inhibitor of nuclear factor of kappa B, alpha IκBα, LC3b, LC3a, phospho-p38^Thr180/Tyrosine (Tyr) 182^, phospho-ULK1^Ser757^, total ULK1 (Cell Signaling Technology, Leiden, The Nederlands), p62 (Progen Biotechnik, Heidelberg, Germany) or proteasome 20S α + β (Abcam, Cambridge, UK). Membranes were washed three times with TBST and incubated for 1 h at room temperature with a secondary antibody conjugated to horseradish peroxidase (Sigma-Aldrich). Membranes were washed three times before detection by chemiluminescence with ECL-Plus Western blotting kit (Amersham Biosciences, Diegem, Belgium). Films were scanned on an ImageScanner using the Labscan software and bands were quantified with the Image Master 1D Image Analysis Software (Amersham Biosciences). Expression levels were normalized to eEF2, whose expression was unaffected by treatments. Western blots for RPT1 and protein carbonyls utilized Pierce Pico plus ECL reagent, and quantification was carried out using Quantity One Analysis Software (Bio-Rad, CA, USA) with Ponceau S total protein staining of the lane as the normalization control as previously described [26]. All the bands detected in each lane by the anti-dinitrophenyl antibody (protein carbonyls) were quantified relative to total protein staining (Ponceau S).

### Protein extraction for enzymatic activities

Muscles were cut into small pieces with razor blades then homogenized on ice with a Tenbroeck Tissue Grinder in ice cold buffer containing 50 mM Tris, pH 7.5, 150 mM NaCl, 5 mM MgCl_2_, 1 mM EDTA and 1 mM DTT. Homogenates were centrifuged for 30 min at 10,000 g, 4°C. Supernatants were stored at -80°C. Protein content was determined using a Bradford protein assay kit (Bio Rad) with BSA as a standard.

### Enzymatic activity assays

#### Proteasomes

Enzymatic activities were determined fluorometrically using specific substrates and inhibitors, as previously described [27,28]. Each sample was assessed in quadruplicate with two replicates containing inhibitors. For each assay, all samples were run on the same plate. 26S proteasome activities were determined by adding 100 μM Z-LLE-AMC (Peptide Institute), LSTR-AMC (Bachem, CA, USA) or Suc-LLVY-AMC (Bachem) for the β1, β2 and β5 subunit activities respectively. Assays using 25 μg of protein were carried out in a reaction buffer containing 50 mM Tris, pH7.5, 1 mM EDTA, 150 mM NaCl, 5 mM MgCl_2,_ 0.5 mM DTT and 100 μM ATP, ± inhibitor (40 μM Z-Pro-Nle-Asp-al (β1) (Biomol, PA, USA), 60 μM epoxomicin (β2) or 20 μM epoxomicin (β5)). 20S proteasome activities were determined similarly but using different reaction buffers: β1 and β2 activities were assayed in 25 mM HEPES, pH 7.5, 0.5 mM EDTA, 0.05% NP-40, 0.001% SDS. The β5 activity was assayed in a similar buffer with the exception that the 0.05% NP-40 and 0.001% SDS were replaced with 0.03% SDS [27,29,30]. All 20S activity measurements were carried out in the absence of ATP but in the presence of detergent [28]. Fluorescence was monitored every 15 min for 115 min on Fluoroskan Ascent FL (Thermo Scientific, MA, USA) at an excitation and emission wavelength of 380 nm and 460 nm, respectively. Enzymatic activity was calculated as the difference between fluorescence intensity in the absence of inhibitor and fluorescence intensity in the presence of inhibitor at 45 min. The fluorescence intensity was linear over a range greater than 60 min.

#### Cathepsins

Cathepsin activities were assessed with 20 μg proteins per well. Cathepsin B activity was assayed with 100 μM Z-Arg-Arg-AMC (Biomol) in a reaction buffer containing 44 mM KH_2_PO_4_, pH 6.0, 6 mM Na_2_HPO_4_, 0.67 mM EDTA, 1.35 mM Cysteine ± 10 μM cathepsin B Inhibitor (Biomol). Cathepsin L activity was determined with 100 μM Z-Phe-Arg-AMC (Peptide Institute) in a buffer containing 100 mM sodium acetate, pH 5.5, 1 mM EDTA, 1 mM DTT ± 10 μM cathepsin L inhibitor I (Calbiochem, Darmstadt, Germany). Fluorescence was also determined at excitation and emission wavelengths of 380 nm and 460 nm as carried out for the proteasome assays.

### Polyubiquitination ELISA Assay

ELISA assays were performed in high binding 96-well microtiter plates (Santa Cruz Biotechnology, CA, USA). Wells were incubated with 1 μg of muscle lysate overnight at 4°C, washed four times with PBST and excess binding sites blocked with PBST containing 5% BSA. Bound polyubiquitinated proteins were detected using an anti-polyubiquitin antibody FK1 (Biomol), which does not bind monoubiquitinated proteins or free ubiquitin. Bound anti-polyubiquitin antibodies were detected using anti-IgG/IgM conjugated with Horseradish peroxidase (Sigma-Aldrich). After 1 h incubation with the secondary conjugate, the plates were washed 5 times in PBST and incubated at room temperature with Sureblue TMB substate (KPL, MD, USA). After 5 min the color development was stopped using 2.5 M sulphuric acid and the color developed recorded with a Bio-Rad 680 microplate reader at 450 nm. Controls using BSA and ubiquitin (negative) and penta polyubiquitin chains (Biomol, positive control) were used to validate the assay conditions.

### RNA extraction and quantitative Real-Time PCR

Powdered muscles were homogenized in 1 ml Trizol® reagent (Invitrogen, Merelbeke, Belgium). RNA was isolated according to the manufacturer’s instructions. RNA quality and quantity were assessed by 1.5% agarose gel electrophoresis and Nanodrop® spectrophotometry. Reverse transcription was performed from 1 μg RNA using the iScript™cDNA Synthesis Kit from Bio-Rad, according to the manufacturer’s instructions. Primers used for quantitative PCR are reported in Table 1. Experiments were performed on MyIQ2 thermocycler, using the following conditions: 3 min at 95°C, followed by 35 cycles of 30s at 95°C, 30s at 60°C and 30s at 72°C. For each gene, all samples were run in triplicate on the same plate. Each reaction was processed in a 10 μl volume containing 4.8 μl IQ SybrGreen SuperMix (Bio-Rad), 0.1 μl of each primer (100 nM final) and 5 μl cDNA of the appropriate dilution. Melting curves were systematically assessed for quality control. Relative mRNA expression levels were normalized using the geNorm method described by Vandesompele et al. [31]. For each sample, a normalization factor was calculated, based on the geometric mean of the two most stable genes out of the five tested (*Cphn*, *Rpl4*).

**Table 1:** Sequences of primers (5’-3’)

**Gene**	**Forward**	**Reverse**	**NCBI accession number**
***Cphn***	CGTCTCCTTCGAGCTGTTTG	CCACCCTGGCACATGAATC	NM_008907.1
***MAFbx***	CCATCAGGAGAAGTGGATCTATGTT	GCTTCCCCCAAAGTGCAGTA	NM_026346.3
***MuRF1***	ACGACATCTTCCAGGCTGCGAATCC	TCTCGTCTTCGTGTTCCTTGC	NM_001039048.2
***Psmb1***	CTTGATGAAGAAGGAAAGGGAG	TGGTTGTCGAGCAGAGGC	NM_011185.3
***Psmc2***	GAAGGACGACAAGCCCATC	AGGTGCCAACCCAGTGTCAG	NM_011188.3
***Rpl4***	CGCAACATCCCTGGTATTACT	TGTGCATGGGCAGGTTATAGT	NM_024212.4
***Tnf-α***	CAGACCCTCACACTCAGATCA	CCTTGTCCCTTGAAGAGAACC	NM_013693.3

### Statistical analysis

Values are presented as means ± SEM. A one-way ANOVA was conducted to evaluate statistical significance, except for body weight on which a two-way repeated measures ANOVA was applied. The Fisher LSD Method was used for post-hoc tests. Statistical significance was set at *P* < 0.05.

## Results

**Figure 1:**
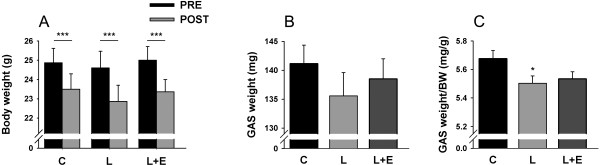
**Animal characteristics.** Body weight **(A)**, GAS weight **(B)** and ratio of GAS weight on body weight (BW) **(C)** of mice 8 hours after administration of LPS 7.5 mg/kg with or without epoxomicin 0.5 mg/kg treatment. Weight of GAS was measured in left and right muscles of control **(C)**, LPS (L) and LPS + epoxomicin (L + E) treated animals. Values are means ± SEM. Panel **A**: *** *p* < 0.001, PRE *vs.* POST; Panel **C**: * *p* < 0.05, compared with **C**.

In this study, we choose an acute model of LPS administration to investigate the modulation of the various components of the UPP as well as the regulatory pathways within the GAS. Due to this acute model, there was no difference between groups in body weight loss, which is likely attributed to food deprivation only (Figure 1A). GAS weight was not significantly modified (Figure 1B). Nevertheless, the GAS weight/body weight ratio (Figure 1C) was 3.05 ± 0.73% lower (P = 0.027) in the L group, compared to the C group.

**Figure 2:**
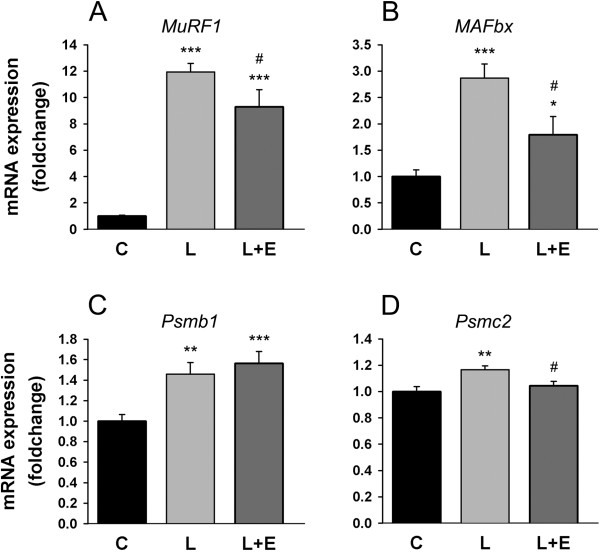
**Transcriptional regulation of UPP.** mRNA expression levels of *MuRF1 ***(A)**, *MAFbx ***(B)**, *Psmb1 ***(C)** and *Psmc2 ***(D)** in the GAS of mice 8 hours after administration of LPS 7.5 mg/kg with or without epoxomicin 0.5 mg/kg treatment (L + E and L, respectively). Values are means ± SEM. * *p* < 0.05, ** *p* < 0.01, *** *p* < 0.001 compared with control **(C)**; ^#^*p* < 0.05 compared with LPS (L).

The timing and the dose of LPS selected are known to drastically increase *MuRF1* and *MAFbx* expression [7], which are considered to be master regulators of UPP-related proteolysis in skeletal muscle [25]. In agreement with the literature, *MuRF1* and *MAFbx* mRNA levels were increased after LPS administration by 11.93 ± 0.66 fold (P < 0.001) and 2.87 ± 0.26 fold (P < 0.001), respectively (Figure 2A,B). A smaller increase was observed for the proteasome subunits *Psmb1* (1.46 ± 0.12 fold, P = 0.006) and *Psmc2* (1.17 ± 0.03 fold, P = 0.003) (Figure 2C,D). Epoxomicin administered 1 h before LPS repressed the increase in *MuRF1* by 24% (P = 0.044), *MAFbx* by 58% (P = 0.010) and *Psmc2* by 74% (P = 0.018) (Figure 2A,B,D).

**Figure 3:**
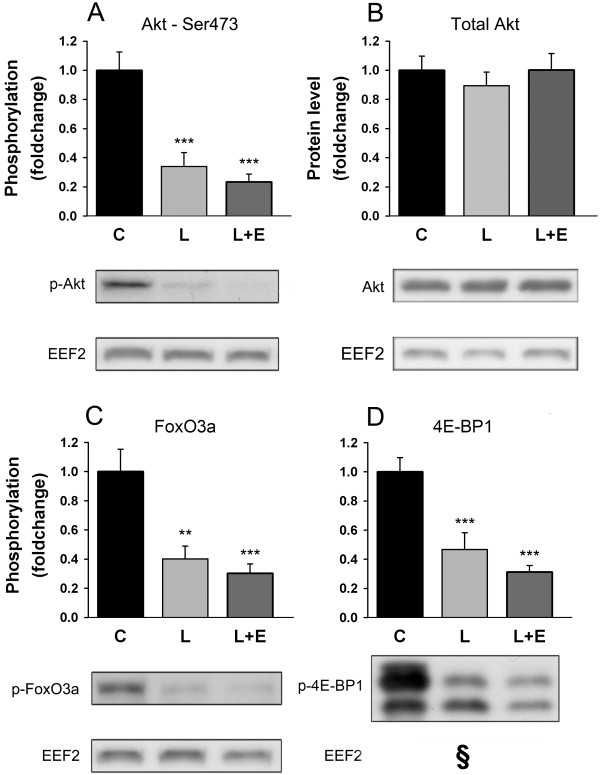
**Phosphorylation state of UPP regulatory proteins.** Phosphorylation state of Akt^Ser473^**(A)**, protein level of Akt **(B)**, phosphorylation states FoxO3a^Thr32^**(C)** and 4E-BP1^Thr37/46^**(D)** in the GAS of mice 8 hours after administration of LPS 7.5 mg/kg with or without epoxomicin 0.5 mg/kg treatment. Values are means ± SEM. ** *p* < 0.01, *** *p* < 0.001 compared with control **(C)**. ^§^: Western blot analyses for 4E-BP1 ^Thr37/46^ and FoxO3aThr32 originate from the same gel and the representative western blot comes from the same set of samples. Therefore, loading control eEF2 is the same.

Amongst the transcription factors implicated in the control of atrogene expression, members of the forkhead box O (FoxO) family, such as FoxO3a seem to be master regulators [32]. In atrophying muscles, Akt is known to be dephosphorylated, leading to subsequent dephosphorylation of FoxO3a which can in turn translocate into the nucleus and activate transcription of target genes [33]. With LPS, Akt and FoxO3a were dephosphorylated by 66 ± 10% (P < 0.001) and 60 ± 9% (P = 0.002), respectively (Figure 3A,C). As total Akt protein level was constant (Figure 3B), this supports a nuclear translocation of FoxO3a that is dependent on the inactivation of Akt. The latter also controls protein synthesis through the activation of mammalian target of rapamycin (mTOR). We measured the phosphorylation state of a downstream target of mTOR, namely 4E-BP1, which was lowered by 53 ± 11% (P = 0.001) in the L group (Figure 3D). Treating with epoxomicin had no further effect on the phosphorylation states of Akt, FoxO3a and 4E-BP1 in comparison with LPS treatment alone (Figure 3).

**Figure 4:**
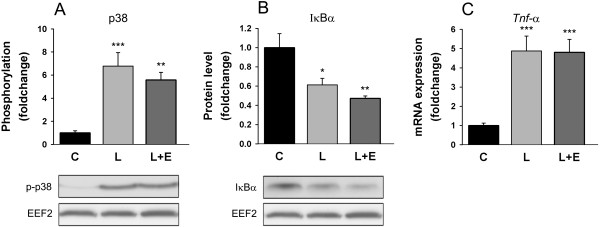
**LPS signaling.** Phosphorylation state of p38^Thr180/Tyr182^**(A)**, protein expression level of IκBα **(B)** and mRNA expression level of *Tnf-α* in the GAS of mice 8 hours after administration of LPS 7.5 mg/kg with or without epoxomicin 0.5 mg/kg treatment. Values are means ± SEM. * *p* < 0.05, ** *p* < 0.01, *** *p* < 0.001 compared with control **(C)**.

LPS is a ligand for toll-like receptor 4 (TLR4) [34]. This membrane receptor is known to activate p38 mitogen-activated protein kinase (MAPK) [21] and nuclear factor kappa B (NF-κB) [35], two pathways implicated in the regulation of atrogene transcription [36-38]. With LPS, p38 phosphorylation increased by 6.78 ± 1.16 fold (P < 0.001), the NF-κB target gene tumor necrosis factor alpha (*Tnf-α*) increased by 4.88 ± 0.78 fold (P < 0.001) and the protein level of IκBα was decreased by 39 ± 7% (P = 0.013) (Figure 4). Again, adding epoxomicin did not have any further effect on phospho-p38, *Tnf-α* or IκBα when compared to LPS alone (Figure 4).

**Figure 5:**
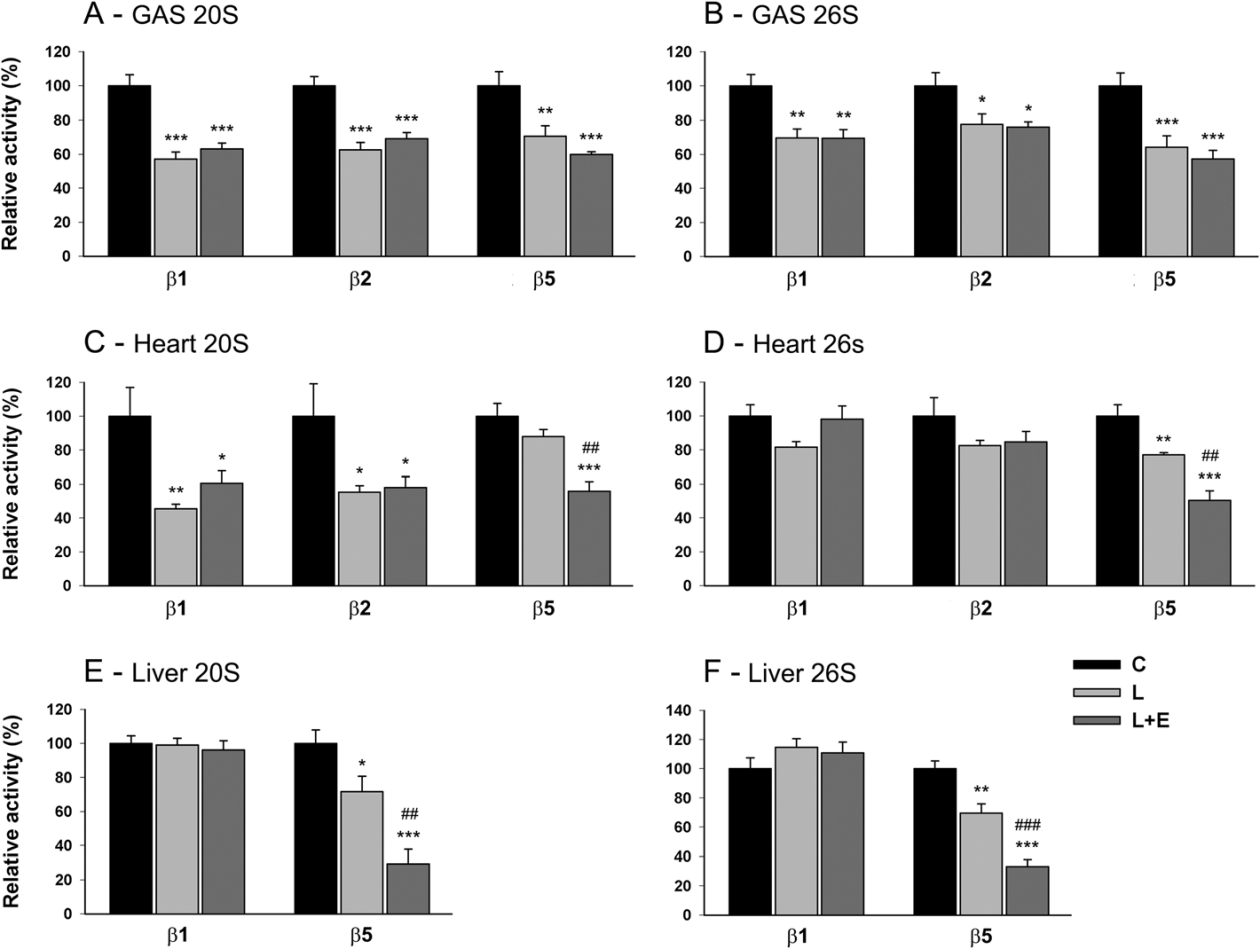
**Effect of LPS on proteasome activities in gastrocnemius, heart and liver.** Proteasome 20S **(A, C, E)** and 26S **(B, D, F)** enzymatic activities in the GAS **(A, B)**, heart **(C, D)** and liver **(E, F)** of mice 8 hours after administration of LPS 7.5 mg/kg with or without epoxomicin 0.5 mg/kg treatment. Values are means ± SEM. * *p* < 0.05, ** *p* < 0.01, *** *p* < 0.001 compared with control **(C)**; ^##^*p* < 0.01, ^###^*p* < 0.001 compared with LPS (L).

Proteasome 20S and 26S enzymatic activities are shown in Figure 5. Epoximicin is a selective proteasome inhibitor, which shows the highest affinity for β5. Nevertheless, in GAS muscle, the β5 activity was not significantly lower in the L + E group compared to the L group (Figure 5A,B). Because of this unexpected result, we measured proteasome activities in heart and liver. As expected, the β5 activities of the 20S and 26S proteasome were lower in the L + E group compared to the L group in liver (-59% and -52%, respectively) and heart (-36% and -35%, respectively; Figure 5C-F).

Since LPS is known for inducing muscle atrophy [39], we expected an increase in proteasome activity after LPS injection. However, the results showed a general decrease in proteasome activities 8 hours after LPS injection. In GAS muscle, LPS induced a decrease in all proteasome activities (Figure 5A,B): -43%, -38% and -29% for the 20S β1, β2 and β5 activities respectively, and -30%, -22% and -36% for the 26S β1, β2 and β5 activities respectively. In heart, 20S β1 (-55%), 20S β2 (-45%) and 26S β5 (-23%) proteasome activities were lower in the L group in comparison with the C group (Figure 5C,D). In liver, the 20S β5 and 26S β5 activities were also decreased (-29% and -30%, respectively; Figure 5E,F). The β2 activity in the liver was not measured because this tissue contains significant non-proteasomal trypsin-like protease(s) that readily cleaves the proteasome substrate, preventing highly accurate proteasomal activity measurements [40].

**Figure 6:**
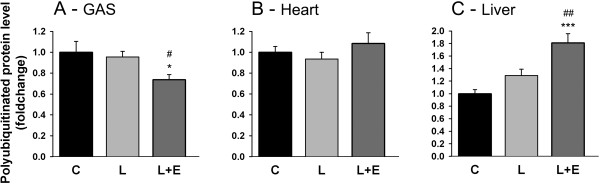
**Levels of steady state polyubiquitinated proteins. (A)**, heart **(B)** and liver **(C)** of mice 8 hours after administration of LPS 7.5 mg/kg with or without epoxomicin 0.5 mg/kg treatment. Values are means ± SEM. * *p* < 0.05, *** *p* < 0.001 compared with control **(C)**; ^#^*p* < 0.05, ^##^*p* < 0.01, compared with LPS (L).

The levels of steady state polyubiquitinated proteins are presented in Figure 6. LPS did not significantly affect polyubiquitination although there was a trend toward increased polyubiquitination in liver (1.28 ± 0.10 fold, P = 0.077, Figure 6C). Epoxomicin decreased polyubiquitination by 23% in GAS muscle (L vs. L + E, P = 0.051, Figure 6A) and conversely increased it by 41% in liver (L vs. L + E, P = 0.004, Figure 6C), the latter being consistent with proteasome β5 inhibition.

**Figure 7:**
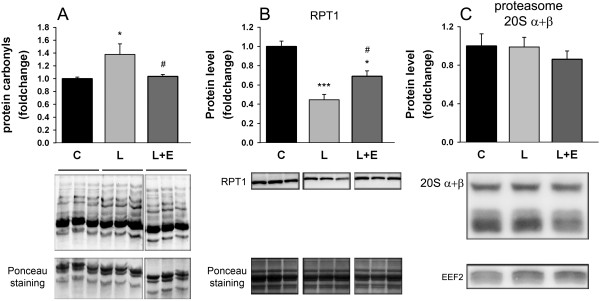
**Markers of LPS-induced oxidative stress.** Effect of LPS on protein carbonyl expression level **(A)**, RPT1 protein expression level **(B)** and proteasome 20S α + β protein expression level **(C)** in the GAS of mice 8 hours after administration of LPS 7.5 mg/kg with or without epoxomicin 0.5 mg/kg treatment. Samples of each blot were ran on the same gel. Values are means ± SEM. * *p* < 0.05, *** *p* < 0.001 compared with control **(C)**; ^#^*p* < 0.05 compared with LPS (L).

RPT’s (proteasome regulatory particles) are ATPases that are located in the 19S proteasome regulator base and which are targets for carbonylation [41]. Figure 7 shows that LPS administration increased carbonyl levels by 38% (P = 0.022, Figure 7A) while the 19S ATPase RPT1 protein levels decreased by 55% (P < 0.001, Figure 7B), both in GAS. When epoxomicin was administrated to LPS animals, the increase in carbonyl levels was completely rescued (L vs. L + E, P = 0.033, Figure 7A) while the decrease in RPT1 was rescued by 44% (L vs. L + E, P = 0.027, Figure 7B). Alternatively, protein levels of proteasome 20S remained unchanged (Figure 7C).

**Figure 8:**
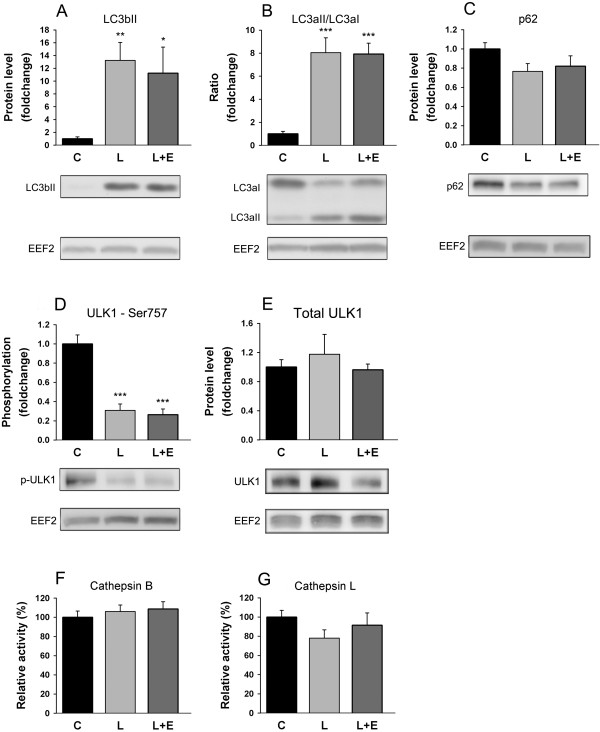
**Effect of LPS on autophagy protein markers and lysosomal enzymes.** Protein expression level of LC3bII **(A)**, protein expression ratio of LC3aII on LC3aI **(B)**, protein expression level of p62 **(C)**, phosphorylation state of ULK1^Ser757^**(D)**, protein expression level of ULK1 **(E)**, enzymatic activities of Cathepsin B **(F)** and Cathepsin L **(G)** in the GAS of mice 8 hours after administration of LPS 7.5 mg/kg with or without epoxomicin 0.5 mg/kg treatment. Values are means ± SEM. * *p* < 0.05, ** *p* < 0.01, *** *p* < 0.001 compared with control **(C)**.

Accumulation of LC3bII has been best correlated to autophagosome accumulation and used as a positive marker for increased autophagosome presence [42], while determination of the ratio of LC3II on the non-lipidated form (LC3I) has been considered a reliable assay for autophagosome synthesis [43]. In GAS, LC3bII and the LC3aII/LC3aI ratio were increased after LPS injection by 13.24 ± 3.09 fold (P = 0.005, Figure 8A) and 8.04 ± 14.41 (P < 0.001, Figure 8B) fold respectively. It was not possible to calculate an LC3bII/LC3bI ratio as the antibody (catalog number 3868, Cell Signaling Technology) that we used has a stronger reactivity with the LC3bII form than with the LC3bI form [21]. The LPS-induced changes in LC3 were not altered when epoxomicin was administered. Autophagosome accumulation inside the cell can be due to defects in lysosomal degradation that can be assessed via the accumulation of p62 [44,45], which is a protein cargo involved in degradation of ubiquitinated protein aggregates through autophagy and which is known to be degraded together with autophagosome content. In GAS, p62 protein levels were lowered by 23% after LPS injection (P = 0.058, Figure 8C). The autophagy inductor ULK1 is repressed when phosphorylated at Ser757 by mTOR. In GAS, phosphorylation of ULK1 at Ser757 was lowered by 69% after LPS injection (P < 0.001, Figure 8D), while the total form of the kinase was unaffected by treatments (Figure 8E). Cathepsin B and L lysosomal enzymatic activities were not affected by LPS administration or treatment with expomicin prior to LPS (Figure 8F and G).

## Discussion

The main finding of the present study is the unexpected attenuation of all proteasome activities in skeletal muscle during the early hours of LPS-induced endotoxemia. The same pattern of regulation was also observed in cardiac tissue while only β5 activities were decreased in liver. As detailed in the methods, enzymatic activities were determined fluorometrically using specific substrates and inhibitors, following a validated protocol [28] that was previously used to show increase in proteasome activities following denervation-induced muscle atrophy [27].

The regulation of proteasome activity in skeletal muscle in response to LPS administration was investigated by previous *in vivo* studies. An increased β5 activity of rat *soleus* and *extensor digitorum longus* was observed 24 hours after LPS for doses ranging from 1 to 12 mg/kg [17,20] and similar results were reported for the diaphragm of rat 48 hours after LPS administration [18]. Intravenous administration of a low dose of endotoxin to human reduced protein degradation without changing proteasome β5 activity after 3 hours [19]. Taken together, these results suggest that different rodent models (rat or mice) as well as the delay between LPS injection and muscle collection might explain the discrepancy between our results and those previously reported in the literature. Therefore, the choice of the delay between LPS injection and the animal sacrifice is critical. We choose a delay of 8 hours because this delay was known to drastically increase *MuRF1* and *MAFbx* expression [7], which are considered to be master regulators of UPP-related proteolysis in skeletal muscle [25].

The mechanisms responsible for the decrease in proteasome activities in the early hours after LPS injection remain unclear. This decrease was not related to changes in proteasome 20S abundance, as the various 20S subunits, including β1, β5 and β5i were not affected. Nevertheless, these subunits must be assembled to fulfill their degradation function. Hence, it is possible that proteasome assembly was altered by acute LPS administration without showing any changes in protein subunit expression levels. Further research should be conducted to investigate that hypothesis. Alternatively, proteasome 20S activity is regulated through association with regulatory particles, the best described of them being the proteasome 19S. A reduction in proteasome activities has been proposed as a mechanism for sparing energy since less ATP would be consumed by proteasome 19S [46]. The reduced level of RPT1 ATPase due to sepsis is an element in favor of that hypothesis, which is also supported by the close association between endotoxemia and a decreased ability to generate ATP through oxidative metabolism during acute phase of sepsis [47].

Proteasome assays measure the catalytic activity of the 20S (ATP-independent) or 26S forms (ATP-dependent) for a given substrate concentration. Our results suggest the presence of an inhibitory process, which represses all proteasome activities in response to acute LPS administration. Oxidative stress could be the inhibitory link between LPS and proteasome inhibition. Proteasomal degradation is known to be repressed *in vitro* through carbonylation of ATPase subunits of proteasome 19S [41]. 19S ATPases – also known as Rpts - are located in the base of the 19S particle. Their functions are to bind substrates selectively, to open the gate formed by the α-ring of the 20S, to unfold substrates and to allow substrate translocation inside the proteolytic room of the 20S. For all these reasons, their association with the 20S proteasome stimulates proteasomal protein degradation [48]. In this study, we showed that the level of protein carbonyls increased with LPS administration and was associated with a large decrease in RPT1 protein level. This coincides with the decrease in proteasome 20S activities. As carbonyls can be degraded independently of ATP directly by the 20S, this supports a role of oxidative stress in LPS-induced proteasome inhibition.

Transcripts coding for proteasome subunits PSMB1 and PSMC2 were increased 8 hours after LPS administration. This suggests that a feed-back mechanism occurs to restore/increase the level of proteasome 19S/20S subunits, which is consistent with the later increase in proteasome activity reported by other investigators [17,18,20].

Even though the catalytic activities of the proteasome were decreased, the total protein amount was likely lower after LPS administration [6]. Indeed, the GAS/body weight ratio was decreased supporting the idea that atrophy was already occurring at the time of the sacrifice, i.e. 8 hours after LPS injection. The decrease in the assayed proteasome activities does not necessary implicate an *in vivo* repression of the overall UPP activity. A higher amount of substrate available for proteasome degradation could increase the rate of protein breakdown through the proteasome. Although the amount of protein available for 20S proteasome degradation is unknown, the lack of any change in the levels of steady state polyubiquitinated proteins after LPS injection argues against a substrate-dependent regulation of the 26S form. It is possible that although the proteasome activities are reduced by LPS, they remain high enough to ensure the removal of ubiquinated substrates available for degradation *in vivo*. Taken together, our results clearly show that UPP components are not necessarily regulated in the same way in response to a catabolic signal such as LPS and suggest that mechanisms of retro-feedback could occur to prevent any excessive protein breakdown.

Moreover, increased activity of other degradation pathways like autophagy might also explain the apparent discrepancy between the reduced proteasome activities and the decrease in muscle weight. Indeed, we observed changes in autophagy induction marker phospho-ULK1^Ser757^, autophagosome presence marker LC3bII and autophagosome formation marker LC3aII/LC3aI as well as autophagic flux marker p62, which are all consistent with a strong activation of protein degradation through the autophagy-lysosomal pathway in response to LPS injection, even if cathepsin L and cathepsin B activities were not affected.

A decrease in protein synthesis was observed by Lang et al. 4 hours after LPS administration and was associated to a massive dephosphorylation of 4E-BP1 [6]. Our results also show that LPS induces a dephosphorylation of 4E-BP1 8 hours after LPS injection and reinforce the arguments for a negative protein balance.

Muscle atrophy is a major health matter. Development of therapeutic strategies aiming to counteract muscle loss is crucial. When physical exercise is infeasible and when nutritional strategies are ineffective, the use of pharmacological agents is the only way to prevent muscle atrophy. As UPP is one of the two main pathways responsible for the degradation of the bulk of the proteins in skeletal muscle, proteasome inhibitor administration seems an interesting approach to prevent muscle wasting, especially knowing that a proteasome inhibitor has been approved for hematological malignancy therapy. Additionally, administration of proteasome inhibitors in rodents reduced plasma cytokine increase and prolonged survival in septic shock, which makes them attractive therapeutic agents [13,14]. Epoxomicin was chosen because of its specific inhibition on proteasome activity and its high affinity for the β5 subunit [16], which is believed to be the rate limiting step for proteasomal degradation [12]. Our results showed that *in vivo* administration of epoxomicin was effective for inhibiting 20S and 26S proteasome β5 activities in the hepatic and cardiac muscle cells whereas this inhibition was much weaker and not significant in the skeletal muscle cell. The unexpected inhibition of proteasome activity due to acute LPS administration made it unlikely that proteasome inhibition would have an effect on muscle atrophy under the conditions investigated. A lower sensitivity to proteasome inhibitors inherent to skeletal muscle cells could be due to tissue specific reactivity. Protein degradation was reported to be less sensitive to proteasome inhibitors in isolated skeletal muscles than in cultured cells possibly due to slower up-take or a faster degradation in skeletal muscle than in other tissues [49]. Alternatively, epoxomicin was administered by intraperitoneal injection, a commonly used route for small laboratory animals. Drugs administered intraperitoneally are primarily absorbed through the portal circulation. Therefore, they must pass through the liver before reaching other organs [50]. The liver plays numerous important physiological roles, including detoxification. It is therefore conceivable that epoxomicin was partly removed before reaching the inferior vena cava and being distributed to other tissues. The fact that proteasome activity was strongly inhibited in the liver and to a lesser extent in the heart is an element in favor of that hypothesis. Intravenous and subcutaneous injections are two approved routes of administration of the proteasome inhibitor bortezomib in humans and inhibit 20S proteasome activity to the same extent [51]. Future animal studies could use one of these two routes to be able to correlate the results with human studies.

The results of the present study confirm that the expressions of muscle specific ligases *MuRF1* and *MAFbx* are repressed by proteasome inhibitors. In a previous study, we showed that the administration of another less specific proteasome inhibitor MG132 reduced muscle atrophy caused by a 6-day hindlimb suspension [52]. This was associated with a repression of the increase of *MuRF1* and *MAFbx*. Another study reported similar results in response to a 7-day hindlimb immobilization protocol [53]. Among signaling pathways regulating muscle ligase mRNA expression, NF-κB is a transcription factor potentially repressed by proteasome inhibitor. Under atrophy signal, the inhibitory protein of NF-κB, IκBα becomes phosphorylated and so is marked for ubiquitination and subsequent proteasomal degradation. Therefore, proteasome inhibitors could prevent IκBα degradation and thereby NF-κB activation as well as subsequent increase in ligase mRNA. In our previous study, IκBα was unchanged at the end of the 6-day hindlimb suspension protocol. However, this does not preclude an activation of NF-κB as IκBα may have been degraded at the onset of the unloading and returned to a basal level at the time of sample collection, while *MuRF1* and *MAFbx* remained elevated. Caron et al. also showed a repression of the increase in *MuRF1* and *MAFbx* in immobilized animals treated with MG-132 which was associated to a reduced increase of the *Tnf-α*, *interleukin-6* and *interleukin-1* cytokines, which are known to be regulated by NF-κB [53]. The activation of the NF-κB pathway assessed in the present study by a decreased IκBα level and an increased *Tnf-α* in response to LPS, was not altered by epoxomicin administration. This is consistent with the fact that proteasome β5 activity was not inhibited in skeletal muscle.

Circulating pro-inflammatory cytokines – especially TNF-α - can regulate *MuRF1* and *MAFbx* through p38 activation. Therefore, another explanation for a decrease in muscle ligase mRNA expression could be an anti-inflammatory effect of epoxomicin. However, the lack of change in *Tnf-α* and in p38 phosphorylation state after epoxomicin injection argues against this hypothesis.

Among signaling pathways regulating catabolism, the phosphoinositide-3-kinase (PI3K)/Akt/FoxO3 pathway coordinately regulates UPP and autophagy [54]. Studies dealing with LPS provide controversial results on the activation of this pathway. While LPS increases Akt phosphorylation state in C2C12 cell culture [6], LPS administration *in vivo* seems to repress Akt [21,39]. Here we show a drastic dephosphorylation of both Akt and FoxO3 with LPS administration, which was not reversed by epoxomicin treatment.

Muscle ligase expression can also be regulated by oxidative stress [55]. In the present study, protein oxidation was rescued in LPS animals following epoxomicin administration suggesting indirect anti-oxidant properties for epoxomicin, which could explain the lower increase in *MuRF1* and *MAFbx.*

The main limitation of this study is the lack of a group receiving epoxomicin only. Based on available literature we hypothesized an increase in proteasome activity after LPS injection. The number of groups (3) was chosen with the goal of studying the protective effect of epoxomicin. Due to the LPS-induced decrease in proteasome activities, it is difficult to interpret if epoxomicin was ineffective in muscle because of tissue insensitivity or if epoxomicin is not useful at this time-point because proteasome activities are lowered. To investigate the various components of UPP and the regulatory signaling pathways, we chose an early time-point for sample collection. Therefore, the experimental design used in this study does not allow an investigation of any potential interaction between LPS and epoxomicin.

## Conclusions

In conclusion, the results of the present study show that proteasome activities are reduced in mice skeletal muscle during the acute phase of LPS-mediated endotoxemia whereas mRNA coding for atrogenes is increased. *MuRF1*, *MAFbx* and RPT1 are partially rescued when epoxomicin injection precedes LPS administration. Nevertheless, our data does not support the idea that epoxomicin could be useful for preventing muscle wasting in the early hours of sepsis since muscle mass does not seem to be protected and proteasome activities are not further reduced.

## Abbreviations

4E-BP1: Factor 4E binding protein 1; ATG: Autophagy related-gene; BSA: Bovine serum albumin; C: Control group; DMSO: Dimethyl sulfoxide; DTT: 1,4-dithiothreitol; ECL: Enhanced chemiluminescence; EDTA: Ethylene-diamine tetraacetic acid; eEF2: Eukaryotic elongation factor 2; EGTA: Ethylene-glycol tetraacetic acid; ELISA: Enzyme Linked ImmunoSorbent Assay; FoxO: Forkhead box O; GAS: Gastrocnemius; HEPES: 4-(2-Hydroxyethyl)piperazine-1-ethanesulfonic acid; IκBα: Inhibitor of nuclear factor of kappa B, alpha; L: LPS treated group; L + E: LPS plus epoxomicin treated group LC3, microtubule-associated protein 1 light chain 3; LC3II: Lipidated form of LC3; LPS: Lipopolysaccharide; MAFbx: Muscle atrophy F-box; MAPK: Mitogen-activated protein kinase; mTOR: Mammalian target of rapamycin; MuRF1: Muscle ring finger 1; NF-κB: Nuclear factor kappa B; PBST: Phosphate-buffered Saline with 0.1% Tween 20; PCR: Polymerase chain reaction; PVDF: Polyvinylidene fluoride; Psmb1: Proteasome Subunit Beta Type 1; Psmc2: Proteasome Subunit ATPase 2; RPT1: Regulatory particle triple-A ATPase 1; SDS-PAGE: Sodium dodecyl sulfate polyacrylamide gel electrophoresis; Ser: Serine; TBST: Tris-buffered saline with 0.1% Tween 20; Thr: Threonine; TLR4: Toll-like receptor 4; Tnf-α: Tumor necrosis factor alpha; Tyr: Tyrosine; ULK1: Unc 51-like kinase 1; UPP: Ubiquitin proteasome pathway.

## Competing interests

The authors declare that they have no competing interests.

## Authors’ contributions

CJ: experiment design, collection of samples, analyses, interpretation of data, drafting and revising the manuscript/AG: experiment design, analyses, interpretation of data, revising the manuscript/SD: analyses/LD: experiment design, revising the manuscript/JMR: experiment design, collection of samples, interpretation of data/MF: experiment design, interpretation of data, general supervision and coordination of the study, revising the manuscript/All authors read and approved the final manuscript.

## Pre-publication history

The pre-publication history for this paper can be accessed here:

http://www.biomedcentral.com/1471-2474/15/166/prepub
